# Actigraphic patterns, impulsivity and mood instability in bipolar disorder, borderline personality disorder and healthy controls

**DOI:** 10.1111/acps.13148

**Published:** 2020-01-20

**Authors:** N.M. McGowan, G.M. Goodwin, A.C. Bilderbeck, K.E.A. Saunders

**Affiliations:** ^1^ Department of Psychiatry University of Oxford Oxford UK; ^2^ Oxford Health NHS Foundation Trust Warneford Hospital Oxford UK; ^3^ NIHR Oxford Health Biomedical Research Centre Oxford UK

**Keywords:** borderline personality disorder, bipolar disorder, actigraphy, impulsivity, mood instability

## Abstract

**Objectives:**

To differentiate the relation between the structure and timing of rest‐activity patterns and symptoms of impulsivity and mood instability in bipolar disorder (BD), borderline personality disorder (BPD) and healthy controls (HC).

**Methods:**

Eighty‐seven participants (31 BD, 21 BPD and 35 HC) underwent actigraph monitoring for 28 days as part of the Automated Monitoring of Symptom Severity (AMoSS) study. Impulsivity was assessed at study entry using the BIS‐11. Mood instability was subsequently longitudinally monitored using the digital Mood Zoom questionnaire.

**Results:**

BPD participants show several robust and significant correlations between non‐parametric circadian rest‐activity variables and worsened symptoms. Impulsivity was associated with low interdaily stability (*r* = −0.663) and weak amplitude (*r* = −0.616). Mood instability was associated with low interdaily stability (*r* = −0.773), greater rhythm fragmentation (*r* = 0.662), weak amplitude (*r* = −0.694) and later onset of daily activity (*r* = 0.553). These associations were not present for BD or HCs. Classification analysis using actigraphic measures determined that later L5 onset reliably distinguished BPD from BD and HC but did not sufficiently discriminate between BD and HC.

**Conclusions:**

Rest‐activity pattern disturbance indicative of perturbed sleep and circadian function is an important predictor of symptom severity in BPD. This appears to validate the greater subjective complaints of BPD individuals that are sometimes regarded as exaggerated by clinicians. We suggest that treatment strategies directed towards improving sleep and circadian entrainment may in the future be investigated in BPD.

**Significant Outcomes**

- Objectively determined rest‐activity patterns are strongly correlated with worsened symptoms of impulsivity and longitudinally monitored mood instability in BPD.
- Sleep and circadian features may impact on the chronicity of symptoms in BPD above BD and HCs.
- Future studies targeting rest‐activity pattern modification in BPD are indicated.

**Limitations**

- Sample heterogeneity present for gender and number of unemployed individuals.
- Relative small sample size restricts stratification for medication and comorbid diagnoses.

## Introduction

Bipolar disorder (BD) and borderline personality disorder (BPD) are psychiatric disorders that share overlapping core symptoms of impulsivity and mood instability. Impulsivity, broadly defined, comprises a behavioural pattern of disinhibition, reward‐seeking and action without forethought 1. In both conditions, impulsivity is associated with deleterious outcomes such as increased aggression, substance abuse, self‐harm and acts of suicide 2, 3, 4, 5. Mood instability is characterised by bursts of intense affect and marked difficulties in regulating mood and behaviour 6. In BD and BPD, poorly managed mood instability predicts poorer prognosis, greater number of hospital admissions and suicidal behaviour 7, 8, 9. Thus, explicit treatment of impulsivity 10 and stabilisation of mood 11 should both be considered targets for improvement.

Sleep disturbance is a widely reported sequela of several psychiatric disorders and a suggested transdiagnostic contributor to symptom severity and functional disability 12. In both BD and BPD, sleep disturbance is associated with symptomatic relapse 13, 14. Moreover, dysfunctional sleep has been implicated in several maladaptive neurocognitive processes which underwrite mood and impulsive behaviour 15, 16 highlighting sleep as a potential mediating factor and therapeutic target. A key contributor to healthy sleep is functional operation of the circadian clock, which orchestrates the appropriate timing of rest and activity patterns. The clock operates within and between days, to synchronize (‘entrain’) endogenous circadian rhythms with environmental time signals (i.e. ‘zeitgebers’, particularly light). 17 Monitoring of night‐time sleep and the twenty‐four hour rest‐activity pattern, using technologies such as wrist‐worn actigraphy, has become increasingly prominent in psychiatry. Several studies monitoring rest‐activity patterns and circadian biomarkers have suggested that abnormal circadian rhythm function may be a driver of disordered sleep in BD 13, 18, 19, 20. Circadian dysfunction has been postulated for BPD, but has not hitherto been extensively investigated 21.

Recently, we identified desynchronised patterns of heart rate, activity and sleep, in participants with BD and BPD 14 and correspondingly, reported an association between desynchrony and differential mood patterns 22. We also demonstrated that individuals with BPD exhibit rest‐activity patterns that are suggestive of phase delayed circadian clock function compared with stable BD and healthy controls 23. Thus, sleep and circadian rhythm disruption may be longitudinally specific to BPD, because symptom severity is chronic, rather than intermittent, as in BD. However, it is not presently clear to what degree these disturbances are related to symptom severity in BD and BPD, or whether this relationship differs between conditions. Our previous work examined only group‐wise actigraphy differences in this sample, and a limited assessment of heart‐rate/chest‐mounted acceleration signals with mood outcomes over a short‐term sampling period (4 days). To our knowledge, there have been no studies specifically examining how objectively assessed sleep/circadian actigraphy parameters valid over several weeks are related to impulsive symptoms or longitudinal mood instability in BPD.

### Aims of the Study

The current study aimed to investigate the relationship between core symptoms of both BD and BPD with the circadian structure and timing of the actigraphy assessed rest‐activity rhythm. We compared individuals with BD, BPD and healthy volunteers recruited from the general population in order to differentiate symptom‐actigraphy measured associations in those with psychiatric disorder to those without. As impulsivity and mood instability are transdiagnostic features that are core symptoms in both conditions, the principal unelucidated question that we aim to clarify is whether BD and BPD differ in terms of symptom severity relating to sleep/circadian rhythm function.

## Material and methods

### Study design

This study used data from three groups: participants diagnosed with BD, BPD and healthy volunteers, all of who were enrolled in the Automated Monitoring of Symptom Severity (AMoSS) study 24 conducted between March 2014 and February 2016. BD and BPD participants were recruited from out‐patient services around Oxfordshire, UK or from registration lists for other ongoing studies. Healthy volunteers were recruited from the community. Written informed consent was obtained from all participants. All procedures contributing to this work comply with the ethical standards of the relevant national and institutional committees on human experimentation and with the Helsinki Declaration of 1975, as revised in 2008. All procedures were approved by the NRES Committee East of England—Norfolk (13/EE/0288).

### Participants

The data presented here consist of measurements from 87 participants with valid actigraphy recordings: 31 BD (mean age [SD] = 39.2 [12.2] years, 21 females), 21 BPD (34.1 [10.5] years, 19 females) and 35 healthy controls (HC) matched for age (39.5 [12.5] years, 24 females). Extensive demographic details of this sample and data quality control procedures for actigraph data processing have been described previously 23. Demographic and clinical features of the sample are presented in Table S1. Participant diagnoses were confirmed prior to study admission by an experienced psychiatrist (KEAS) using the Structured Clinical Interview for DSM‐IV and the International Personality Disorders Examination (IPDE). Exclusion criteria for HCs were as follows: history of neurological disorder or head injury, history of major psychiatric illness and having a first degree relative with a history of BPD or BD. Exclusion criteria for BD and BPD were comorbidity of each diagnosis. Participants with psychiatric disorder were stable throughout the actigraph recording period with Altman Self‐Rated Mania (ASRM) scale completed weekly indicating no manic episode in BD. Depressive symptoms were assessed weekly during the actigraph recording period using the Quick Inventory of Depressive Symptomatology (QIDS) and were significantly greater in BD relative to HC, and significantly greater in BPD relative to HC and BD. Groups were age matched at study entry but differed in terms of gender composition and employment status (see Table S1).

### Actigraphy

Monitoring of rest‐activity patterns commenced on the first day participants were enrolled in the study. Participants wore GENEActiv Original actigraphs (ActivInsights Ltd., UK) which were worn continuously on the non‐dominant wrist for 28 consecutive days. The actigraphs contained triaxial accelerometers, which recorded and logged movement throughout the monitoring period at a sampling frequency of 25 Hz. Groups did not differ in terms of actigraph compliance, season when recording took place or the proportion of weekdays or weekend days in each record 23. Exported data were analysed using the validated *GGIR* dedicated package 25 for R version 3.4.2 (R Core Team, Vienna). *GGIR* processes multi‐day raw accelerometer signals and generates standard parameters used to quantify the circadian rhythmicity of rest‐activity patterns previously described by Van Someren et al (non‐parametric circadian rhythm analysis) 26. The following rhythm parameters were derived: interdaily stability, intradaily variability, relative amplitude, L5 and M10 activity levels and onsets and are described below.

Interdaily stability (IS) indicates how stable rest‐activity patterns are between days and therefore reflects the day‐to‐day consistency of behavioural routines. It is adversely affected by behaviours and schedules which result in recurring circadian misalignment.

Intradaily variability (IV) indicates how fragmented states of rest and activity are within the 24 h day and thus may be considered a measure of the consolidation of continuous sleep and wake states. Lower IV values for example emerge from frequent awakenings during night‐time sleep episodes or taking several naps during the day when one would normally be awake.

Relative amplitude (RA) indicates the amplitude or robustness of daily rest‐activity rhythms. It measures the difference in mean activity levels over the most active consecutive 10 h period (M10) compared with the least active consecutive 5 h period (L5) of the 24 h pattern and is expressed in relative terms for each individual. Lower RA reflects little differentiation between periods of rest and activity and therefore a weaker circadian rhythm of the rest‐activity pattern. It is associated with poor circadian entrainment to oscillating *zeitgebers*.

M10 and L5 provide additional information about arousal patterns during the day and night. For example, lower M10 values correspond with inactivity during the day while higher L5 values suggest disturbed sleep. M10 onset and L5 onset times indicate phase markers of circadian function corresponding with the onset of activity during the day and the offset of activity in during the night. Activity assessed in this manner is a validated measure of circadian phase predicting the circadian rhythm of melatonin secretion 27. The standard deviation of daily M10 and L5 activity patterns and onset times, and RA were also calculated to provide additional information on variability of activity, phase, and amplitude during the recording period.

### Assessments

#### Barratt Impulsiveness Scale

The Barratt Impulsiveness Scale (BIS‐11) 28 was used to assess impulsivity. Self‐reported ratings on 30 questions measuring three domains of impulsivity (‘attentional impulsiveness’, ‘motor impulsiveness’ and ‘non‐planning impulsiveness’) were used to determine a total BIS‐11 score which can range from 30 to 120. Higher scores indicate greater levels of trait impulsivity.

#### Mood Zoom

The Mood Zoom (MZ) questionnaire was conceived as part of the AMoSS study to provide a compact daily assessment of mood variability delivered via a smartphone application. The MZ comprises six mood items: ‘anxious’, ‘elated’, ‘sad’, ‘angry’, ‘irritable’ and ‘energetic’. Participants were asked each day to rate to what extent the aforementioned words described their current mood on a 7‐point Likert scale ranging from ‘Not at all’ to ‘Very much’. Participants were recruited to use the MZ for an initial 3 month period with the option to remain in the study for 12 months or longer. The mean length of the monitoring period for the total sample was 429 days (SD = 239) and did not differ significantly between groups (Table S2). Median adherence after the full 12 month observation period was> 79% irrespective of diagnosis or HC status, displaying a robust acceptability of the instrument 24.

A three‐component structure of the MZ questionnaire has previously been described summarising ‘positive’, ‘negative’ and ‘irritability’ factors which correlate well with standardised measures of depression (QIDS), anxiety (GAD‐7) and mental health status (EQ‐5D) 24. To assess mood instability, we extracted cross‐sectional statistics from each participant’s longitudinal data set of daily MZ ratings. We used the root mean square of successive differences (RMSSD) to measure mood instability, which is a measure of variability reflecting both the temporal order and amplitude of the data 29. Marked differences in mood variability between these groups using the RMSSD operator have previously been described 24. In the supplementary material, we report comparisons with actigraphic variables using differential assessments of variability: standard deviation (SD), Teager–Kaiser Energy Operator (TKEO) and Shannon entropy. The mean of the z‐score transformed RMSSD of each component was used to measure overall mood instability for each individual during the study observational period.

### Statistical analysis

Partial Pearson product moment correlation coefficients between actigraph parameters and symptom outcomes, controlling for gender and employment status, were analysed for each diagnostic group (i.e. HC, BD or BPD). Covariates inserted were planned in advance given our previous work indicating differences in these characteristics between these groups 23. Distribution of data was inspected using Kolmogorov–Smirnov tests for normality. Observations deviating from normal distribution were first log transformed but non‐normality persisted so partial Spearman’s rank‐order correlation was used. Group‐wise analysis using Fisher’s r to z transformation was used to confirm differences in correlation strength between diagnostic groups and healthy controls. Additionally, we present an exploratory re‐analysis of our previous findings 23 examining whether a classification analysis could discriminate between diagnoses using actigraph measures. The area under the receiver operating characteristic (ROC) curve (AUC) was used to visually inspect the relation between sensitivity and specificity. The Youden Index (i.e. highest value obtained when calculating sensitivity + specificity – 1) was used to determine optimal cut‐off values from which the positive prediction value (PPV) and negative prediction value (NPV) were determined. All analyses were performed in SPSS (IBM) and R version 3.4.2 (R Core Team, Vienna). A significance threshold of *P* < 0.05 was used for all comparisons, and correction for multiple comparisons was applied using the Benjamini–Hochberg false discovery rate (FDR).

## Results

**Figure 1:**
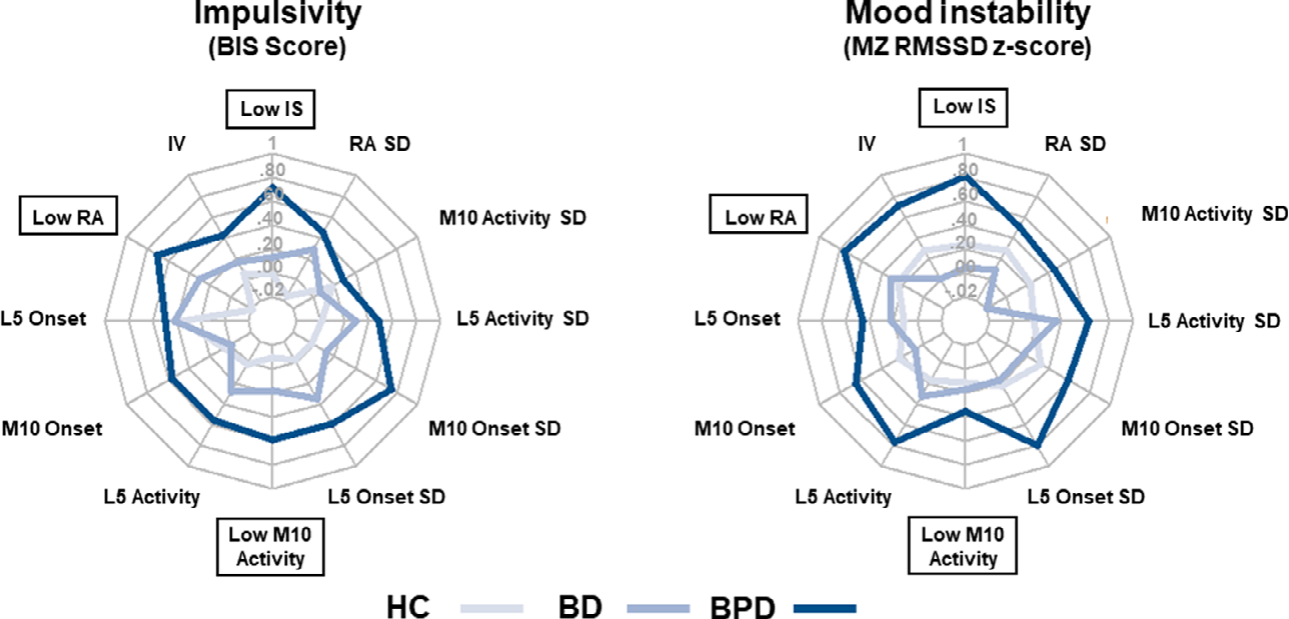
Correlation between sleep/circadian rhythm parameters and symptoms. Radar plots indicate partial correlation coefficient strength between rest‐activity pattern variables and symptoms of impulsivity and mood instability for healthy controls and diagnostic groups. BPD consistently show stronger association between symptoms and parameters indicating perturbed and delayed rest‐activity profile. Negative correlations (e.g. for interdaily stability, relative amplitude and M10 activity) were reversed and renamed for visual consistency such that higher values uniformly indicate stronger association. Labels enclosed in boxes indicate a negative relation. [Colour figure can be viewed at http://www.wileyonlinelibrary.com/]

**Figure 2:**
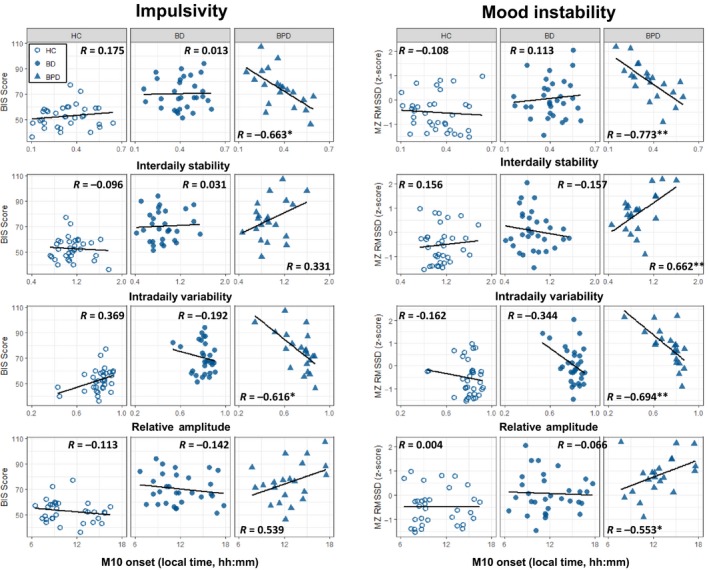
Scatter plots indicating the relationship between sleep/circadian rhythm parameters and symptoms. Scatter plots representing the partial correlation analysis conducted between rest‐activity variables (IS, IV, RA and M10 onset) and impulsivity and mood instability are depicted for each group (HC, BD and BPD). * indicates *P* < 0.05; ** indicates *P* < 0.01. All *P*‐values are adjusted for FDR as per Table S4. [Colour figure can be viewed at http://www.wileyonlinelibrary.com/]

A summary of correlation results are depicted in Figure 1. All analyses described here are partial correlations controlling for gender and employment status (bivariate correlations without covariates are reported in Table S3; partial correlations including covariates are reported in Table S4). Correlation strengths did not appreciably differ between bivariate and partial approaches. Inspection of partial correlation coefficients with covariates inserted revealed strong associations between sleep/circadian rhythm parameters and symptoms of impulsivity and mood instability in BPD. These associations were not found in either the BD participants or HCs. Specifically, in BPD but not HC or BD, rhythm stability (IS) and rhythm amplitude (RA) were negatively correlated with both impulsivity (IS: *r* = −0.663, *P* = 0.012, RA: *r* = −0.616, *P* = 0.020) and mood instability (IS: *r* = −0.773, *P* = 0.001, RA: *r* = −0.694, *P* = 0.006). Thus, less stable and lower amplitude rest‐activity patterns were associated with greater symptom severity. Furthermore, mood instability in BPD but not HC or BD was associated with greater rhythm fragmentation (IV: *r* = 0.662, *P* = 0.006), later onset of daily activity (M10 onset: *r* = 0.553, *P* = 0.028) and greater nocturnal arousal (L5 activity: *r* = 0.560, *P* = 0.028). Representative scatter plots showing associations for IS, IV, RA and M10 onset are presented in Figure 2. Impulsivity and mood instability were respectively associated with more variable L5 activity and activity offsets in BPD indicated by the standard deviation of L5 activity and L5 onset between days (Table S4).

In order to confirm these associations, we performed group‐wise assessment of partial correlation coefficients which indicated that the strength of the relation between symptoms and rest‐activity parameters was significantly greater for BPD than HC and BD (Table S5). There were no significant differences in the strength of these associations between HC and BD. The associations between rest‐activity pattern structure and timing and mood instability were replicated using SD and TKEO operators of variability (Figure S1; Tables S6 and S7) but were not present for entropy (Figure S1; Table S8).

Next, we performed an exploratory classification analysis to determine whether a bottom‐up approach to actigraphic measures could correctly distinguish BD and BPD from HC and BPD from BD. We examined the classification accuracy of the following actigraph summary variables: IS, IV, RA, L5 onset, M10 onset, L5 activity and M10 activity. ROC curve parameters (sensitivity and specificity) and optimal cut‐off values are summarised in Table S9. The L5 onset of activity showed the best classification performance (AUC = 0.83, good accuracy) between BPD and HC with the highest sensitivity and specificity. An L5 onset cut‐off of 01:35 h had a PPV of 0.75 (i.e. probability that participant scoring later had BPD) and a NPV of 0.83 (i.e. probability that participant scoring earlier was HC). The performance of BPD versus BD was fair (AUC = 0.79); an L5 onset cut‐off of 01:37 h had a PPV of 0.71 (i.e. probability that participant scoring later had BPD) and a NPV of 0.81 (i.e. probability that participant scoring earlier had BD). Classification accuracy of L5 onset between HC and BD did not perform sufficiently better than chance (AUC = 0.52). The performance of the remaining aforementioned actigraph summary variables ranged from <0.5 to 0.68 indicating poor classification accuracy.

## Discussion

Unstable interdaily patterns (low IS) and weakened rhythm amplitudes (low RA) are associated with both impulsivity and mood instability in BPD. Furthermore, mood instability in BPD is associated with greater fragmentation of rest‐activity states (IV), greater nocturnal activity levels (L5) and phase delayed daytime activity patterns (M10 onset). We believe these are the first reported results demonstrating an association between objective measures of sleep and circadian function and longitudinal symptom severity in BPD. These associations were not detectable in BD and HC. Furthermore, group‐wise comparison of correlation strengths further confirms these associations in BPD.

There is little or no previous relevant research in BPD. The most recent meta‐analytic review of the literature examining sleep disturbance primarily identified acute polysomnography (PSG) recordings which are limited to single nights 30. These findings describe greater sleep onset latency, poor sleep efficiency and shorter sleep duration in BPD compared with controls 31, 32, 33. Each of these parameters have previously been associated with mood disturbance 34. However, the longitudinal picture of sleep is not adequately captured by PSG and few studies have assessed core symptoms in parallel with sleep. Actigraphy studies in BPD are sparse, conducted over short intervals and similarly limited regarding additional symptom monitoring 35, 36. Subjective assessments of chronic sleep disturbance have suggested an association with worsened BPD symptoms among community and out‐patient groups 37, 38, 39. The present findings indicate that core symptoms are associated with objectively measured sleep/rest‐activity patterns. Thus, we propose that disturbances in initiating/maintaining sleep may be an important exacerbating factor for borderline psychopathology.

The correlations between worsened symptoms of impulsivity and mood instability with unstructured rest‐activity patterns, characterised by lower relative amplitude, lower interdaily stability and greater phase variability within days, suggest that circadian rhythm disturbance may be an important impairing factor for BPD symptom management. Previous qualitative descriptions of sleep habits of individuals with BPD have emphasised chaotic patterns and poor adherence to socially normal bedtimes 40. Recurring circadian misalignment between habitual bedtimes and social timing imperatives, also termed ‘social jetlag’ 41, may be a feature experienced chronically in BPD. Social jetlag has previously been indicated from actigraphy records in adolescents with BPD 36, and we previously described delayed circadian phase in the current BPD participants 23. Delayed phase is an established risk factor for worsened social jetlag. Such chronic circadian misalignment may lead to non‐optimal organisation of physiologic and cognitive rhythms relative to time‐of‐day demands, so may in turn precipitate impulsive behaviour and mood instability.

The association between impulsivity and alteration of sleep/wake activity is unlikely to be specific to BPD. Indeed, in a previous study of non‐clinical volunteers, higher impulsivity scores were similarly correlated 42. This trait may also be important in ADHD 43. Similarly, mood instability has been associated with lower amplitude rest‐activity patterns but thus far has been measured retrospectively, not prospectively or longitudinally as in our present findings 44, 45. As a core diagnostic feature of BPD, mood instability is, by definition, chronically both extreme and clinically salient 46, 47. The subjective complaints of BPD individuals are sometimes regarded as exaggerated by clinicians. There is regular disagreement between patient reports and assessment by clinicians which are routinely attributed to distorted self‐perceptions and attributions by patients with BPD themselves 48, 49. The stronger correlation with *objective* measures of sleep/wake disturbance can be seen as validating the reported experience of the patients in an interesting way. Individuals with BPD do endorse greater dysfunctional and catastrophic beliefs relating to their sleep, but our data suggest that the impact of sleep‐wake disturbance on symptoms is real and substantial 50.

The association of sleep disturbance and abnormal activity rhythms with BD has been described in multiple systematic reviews of studies employing actigraphy. The most consistent findings in BD are hypersomnia, delayed sleep onset latency and lower sleep efficiency 51, 52, 53. However, differential actigraphy patterns have been described during the manic phase of BD compared with depression, euthymia and healthy controls. These findings highlight that mania is correlated with advanced circadian phase, greater motor arousal and higher frequency variability of rest‐activity patterns 54, 55. Depressed state conversely is associated with delayed sleep phase indicative of delayed circadian rhythm phase of entrainment 56. Our BD participant group recorded median values below 11 on the QIDS and below 6 on AMSR indicating that they were symptomatic but not syndromally depressed or manic 23. Concordant with our classification analysis, we note that none of the actigraphic parameters could reliably distinguish stable BD from HC. The absence of associations between residual symptoms and actigraphy measures further contrasts with the findings for the BPD group.

Mood instability is a transdiagnositc feature common in both disorders. In BPD, it comprises a core diagnostic feature of the disorder typified by rapid alterations in mood over minutes to hours. However in BD, the episodicity of mood symptoms is a principal criterion for diagnosis. Studies involving digital self‐monitoring of mood have reflected these diagnostic differences in the magnitude and frequency of mood alteration. Mood instability between discrete mood episodes in BD is diminished 57 and of lower amplitude than BPD 24. Our measures of mood instability capture short‐term variability through successive observations (RMSSD), general variability of ratings (SD) and instantaneous changes of amplitude and frequency of the signal (TKEO). Thus, we observe associations between clinical symptoms and mood lability that is chronic, intense and rapidly changing and typical for BPD. Therefore, a closer association between disturbed rest‐activity patterns and mood instability in BPD may be expected due to the ecological validity of these measures of variability reflecting interdaily mood vacillation in BPD above BD. Other core symptoms of BPD but not BD, such as severe interpersonal dysfunction and unstable self‐image may also enrich this instability of mood. Our entropy measure was not in the same way associated with actigraphy parameters in either group. Increased entropy, which measures unpredictability, has previously been observed over a shorter window in BD to precede clinically confirmed mood episode 58. Thus, its utility as a measure of instability may be limited to proximal switches in mood state. Future work is indicated in BD to confirm the potential of shorter time‐course measures of mood instability combined with rest‐activity features as predictors of subsequent mood episodes. A further consideration is noted between mood instability and affective instability. While the two terms are often used interchangeably in the literature 6, different conceptualisations exist for each: affect typically refers to the experience of emotion and is short‐lived whereas mood may last longer and lack a focused cause. Clearly, these constructs have implications for symptom measurement, the differentiation of BD/BPD and the contribution of sleep/circadian rhythm disturbance. Greater assessment precision achieved through momentary assessment methods, such as in this study, may in future be bolstered by contemporaneous logging of behaviour and environment in order to clarify such subtleties 11.

Similar to mood, impulsive symptoms differentially expressed between diagnoses may account for the closer relations to sleep/circadian disturbance in BPD. In BPD, impulsive behaviour such as repeated engagement in risky (e.g. reckless decision‐making) or harmful (e.g. self‐injury, parasuicidal) activities is persistent. In BD, behaviours such as risky engagement in pleasurable activities and psychomotor impulsivity (e.g. restlessness/agitation) are diagnostic criteria for mania implying a specific association with mood elevation. Thus, the impulsive modality in BPD is typically chronic and may reflect a stable trait, while in BD it may be intermittent, driven by mood state. Furthermore, differences have been demonstrated in laboratory decision‐making tasks probing impulsivity. Examination of BPD and BD compared with controls show that greater risk‐taking and failure to integrate reward information is a primary deficit in BPD, not in euthymic BD 59. Studies also indicate that current mood status does not prime impulsive neurocognitive responding in BPD 60, 61 as would be expected in BD. These differences may have implications for differential sensitivity to perturbed rest‐activity patterns in BPD over BD. The direction of effect is not apparent. Clearly, disturbed sleep may lead to greater impulsive action 62. Alternatively or additionally maladaptive goal‐directed behaviour and intertemporal perspectives may lead to later bedtimes and irregular circadian entrainment 63. Moreover, the manner in which impulsivity was assessed may further account for different relations between diagnoses. The BIS‐11 is the most widely used instrument for the assessment of impulsivity in clinical populations but a very recent examination of its reliability in euthymic BD suggests the original item structure may not be optimal 64. Although BIS‐11 scores in BD and BPD were similar in the current study (BPD marginally higher, but not significantly different) the instrument may show greater item proximity to the experiences of individuals with BPD compared to BD which may also contribute to the closer associations detected.

The current study goes beyond previously reported group‐wise findings in this sample 23 by examining if symptom severity is associated with sleep/circadian patterns and whether this appears in a diagnostic dependant manner. Our previously reported results raise several implications for the interpretation of findings. Notably, despite a closer association between symptoms and interdaily stability and intradaily variability in BPD, we previously show no differences in group mean score. Relative amplitude was similarly not lower in BPD but there was greater within‐group variability on this measure. As the association with symptoms is at a within‐group level, we suggest a greater reactivity to circadian misalignment and larger proportion of individuals with weak entrainment may drive findings rather than overt between‐group differences. Our previous findings show a delayed rest‐activity pattern in BPD relative to BD and HC through the later onset of L5 and M10 23. Moreover, our classification analysis of actigraph parameters confirmed that later L5 onset discriminated BPD from BD and HC indicating good sensitivity and specificity. However, L5 onset was not significantly associated with symptoms in BPD and M10 onset was only associated with mood instability. Delayed circadian phase also predisposes lower stability and weak rhythm amplitude in the context of poor zeitgeber entrainment. Thus, we further propose that low circadian amplitude and greater misalignment between days in individuals with greater liability to delayed phase (as in BPD) may drive worsened symptoms even if IS and RA differences are not apparent between groups.

The associations described here indicate several translational opportunities for BPD treatment. Stabilisation and consolidation of rest‐activity rhythms might be considered a primary target for adjunctive treatment if indeed symptoms are in part driven by sleep and circadian rhythm disturbances. Preliminary work using bright light therapy in BPD has shown promise resulting in activity phase advance and synchronisation of sleep times 65, and augmentation of antidepressant response when treated with SSRIs 66. Interpersonal and social rhythm therapy (IPSRT) 67 was originally conceived with the purpose of regularising daily patterns in BD so as to mitigate a low threshold for rhythm disruption and subsequent mood decompensation. IPSRT‐inspired approaches may be additionally beneficial in supporting individuals with BPD where such social rhythm instability is apparent. Furthermore, as maladaptive sleep cognitions are common in BPD 50 and this may further perpetuate the severity of insomnia, cognitive behaviour therapy for insomnia (CBT‐I) could be used to address dysfunctional beliefs and behaviours about sleep which has been shown to mediate improvement of depression and psychological well‐being 68, 69.

### Limitations

Although our analyses of between group differences attempted to control for covariates, there was significant heterogeneity of sample demographics between groups, with a preponderance of females and unemployed individuals in the BPD, compared with BD and healthy groups. It is also difficult to control for comorbid diagnoses and divergent medication use in studies of this sample size. Our key findings were at the level of diagnostic group difference, and thus, our approach is limited in providing any within‐subject conclusions. Future studies with greater experience sampling multiple times each day will allow enhanced characterisation of the intradaily dynamics of mood instability within individuals and its relation to adjacent sleep episodes. Further work is also needed to advance the understanding of the relation between sleep and BPD specific symptoms (e.g. interpersonal dysfunction, identity disturbance and abandonment fears) which also explicitly exclude sleep disorders or other comorbid disorders affecting sleep. Although the prospective nature of mood monitoring suggests *prima facie* evidence for long‐term effects of sleep and circadian rhythm disturbance on symptoms, a causal direction of effect has not been established by this study.

To conclude, the current results show a very close association between disrupted sleep and misaligned circadian rest‐activity rhythms and symptoms of impulsivity and mood instability in BPD. An initial 28 day actigraphy record correlated robustly in BPD with impulsivity, and mood outcomes which were prospectively measured over several months. This suggests that untreated rest‐activity rhythm disturbances may associate with worsened clinical course for participants with BPD. Perhaps surprisingly, given the historical interest in BD and circadian function and the symptom overlap with BPD, stable BD patients do not show similar associations. However, small sample sizes limit the generalisability of findings and replication is necessary. In contrast to the evidence showing beneficial outcomes for chronotherapies in BD 70, future studies targeting sleep and circadian rhythm stabilisation in BPD may also produce significant benefits for its treatment.

## Disclosures

Prof Goodwin is a NIHR Emeritus Senior Investigator holds shares in P1vital and P1vital Products and has served as consultant, advisor or CME speaker in the last 3 years for Allergan, Angelini, Compass pathways, Johnson & Johnson, Lundbeck (/Otsuka or/Takeda), Medscape, Minervra, P1Vital, Pfizer, Sage, Servier, Shire, Sun Pharma. Dr. Bilderbeck receives salaries from P1vital Ltd. Dr. McGowan and Dr Saunders report no competing interests.

The views expressed are those of the author(s) and not necessarily those of the NHS, the NIHR or the Department of Health.

## Supporting information


**Figure S1**. Correlation between sleep/circadian rhythm parameters and MZ variability using SD, TKEO and Shannon entropy.
**Table S1**. Summary of demographic and clinical features of sample
**Table S2**. Summary of BIS‐11 and MZ data.
**Table S3**. NPCRA parameter bivariate correlations with impulsivity and mood instability.
**Table S4**. NPCRA parameter partial correlations with impulsivity and mood instability.
**Table S5**. Group‐wise comparison of partial correlation coefficients.
**Table S6.** MZ variability (SD) and NPCRA variables.
**Table S7.** MZ variability (TKEO) and NPCRA variables.
**Table S8.** MZ variability (Entropy) and NPCRA variables.
**Table s9.** Discriminant properties of actigraph measure for diagnosis using ROC curve classification analysis.

## Data Availability

The data that support the findings of this study are available from the corresponding author upon reasonable request.
