# Supplementary material for: Actigraphic patterns, impulsivity and mood instability in bipolar disorder, borderline personality disorder and healthy controls
Source: Acta Psychiatr Scand. 2020 Jan 20;141(4):374–84. doi: 10.1111/acps.13148 (PMC7216871; doi:10.1111/acps.13148)
Supplement: Supplementary file 1 — Figure S1. Correlation between sleep/circadian rhythm parameters and MZ variability using SD, TKEO and Shannon entropy. Table S1. Summary of demographic and clinical features of sample Table S2. Summary of BIS‐11 and MZ data. Table S3. NPCRA parameter bivariate correlations with impulsivity and mood instability. Table S4. NPCRA parameter partial correlations with impulsivity and mood instability. Table S5. Group‐wise comparison of partial correlation coefficients. Table S6. MZ variability (SD) and NPCRA variables. Table S7. MZ variability (TKEO) and NPCRA variables. Table S8. MZ variability (Entropy) and NPCRA variables. Table s9. Discriminant properties of actigraph measure for diagnosis using ROC curve classification analysis. [file ACPS-141-374-s001.pdf]

Supplementary Materials

Acitgraphic patterns, impulsivity and mood instability in bipolar disorder, borderline personality disorder, and healthy controls

Niall M. McGowan [1], PhD; Guy M. Goodwin [1,2], FMedSci; Amy C. Bilderbeck [1], DPhil; and Kate E. A. Saunders [1,2,3], DPhil

Author affiliations:

1 Department of Psychiatry, University of Oxford, Oxford, OX3 7JX, UK

2 Oxford Health NHS Foundation Trust, Warneford Hospital, Oxford, OX3 7JX, UK

3 NIHR Oxford Health Biomedical Research Centre, Oxford, OX3 7JX, UK

Supplementary Table 1: Summary of demographic and clinical features of sample

	HC (*n* = 35)	BD (*n* = 31)	BPD (*n* = 21)	*P*	*Post-hoc*
**Sample Demographics**					
Gender, M:F	11:24	10:21	2:19	0.130	*n.s.*
Age, yr (SD)	39.46 (12.51)	39.23 (12.24)	34.14 (10.5)	0.226	*n.s.*
Unemployed (N)	5	2	11	***P*<0.001**	BPD>HC, BPD>BD
**Clinical Features**					
Bipolar Diagnosis, BD-I:BD-II	-	23:8	-	-	-
IPDE, mdn (IQR)	0 (0.00)	4 (6.25)	16 (3.5)	***P*<0.001**	BD>HC, BPD>HC, BPD>BD
ASRM, mdn (IQR)	0.708 (2.6)	1.5 (3.6)	2.0 (3.53)	0.075	*n.s.*
QIDS, mdn (IQR)	2.0 (2.29)	7.8 (6.0)	13.67 (7.23)	***P*<0.001**	BD>HC, BPD>HC
**Psychotropic medication use, No. (%)**	N/A	30 (97)	16 (76)	***P*=0.023**	BD>BPD
Lithium	-	14	0		
Anticonvulsant	-	6	1		
Antipsychotic	-	22	5		
Antidepressant	-	10	16		
Hypnotic	-	2	1		
Anxiolytic	-	3	5		

HC healthy control, BD bipolar disorder (BD-I, bipolar type I; BD-II, bipolar type II), BPD borderline personality disorder, ASRM Altman Self-Report Mania Scale, IPDE International Personality Disorder Examination module, QIDS Quick Inventory of Depressive Symptomatology. Significant values at P<0.05 are in bold. n.s. indicates non-significant for post-hoc tests. SD standard deviation, IQR interquartile range.

Supplementary Table 2: Summary of BIS-11 and MZ data

	HC	BD	BPD	*P*	*Post-hoc*
**BIS-11**					
BIS-11 (Total)	52.53 (8.74)	70.07 (12.02)	75.33 (15.56)	***P*<0.001**	BD>HC; BPD>HC
BIS-11 (Attention)	12.62 (3.2)	18.7 (4.2)	20.86 (3.85)	***P*<0.001**	BD>HC; BPD>HC
BIS-11 (Motor)	20.56 (3.36)	25.4 (5.45)	26.48 (7.38)	***P*<0.001**	BD>HC; BPD>HC
BIS-11 (Non-Planning)	19.35 (4.04)	25.97 (5.39)	28 (6.95)	***P*<0.001**	BD>HC; BPD>HC
**Mood Zoom**					
Monitoring period, days mean (SD)	383 (238)	479 (285)	430 (226)	0.266	*n.s.*
Negative PCA $_{RMSSD}$ (SD)	1.05 (0.67)	1.65 (0.79)	2.41 (0.8)	***P*<0.001**	BD>HC; BPD>HC; BPD>BD
Positive PCA $_{RMSSD}$ (SD)	1.05 (0.54)	1.27 (0.54)	1.71 (0.54)	***P*<0.001**	BPD>HC; BPD>BD
Irritable PCA $_{RMSSD}$ (SD)	0.68 (0.41)	1.07 (0.42)	1.45 (0.49)	***P*<0.001**	BD>HC; BPD>HC; BPD>BD

HC healthy control, BD bipolar disorder, BPD borderline personality disorder, BIS-11 Barratt's Impulsiveness Scale, SD standard deviation, RMSSD root mean of square of successive differences. Significant values at P<0.05 are in bold. n.s. indicates non-significant for post-hoc tests.

Supplementary Table 3. NPCRA parameter bivariate correlations with impulsivity and mood instability

	**HC**				**BD**				**BPD**			
	**BIS-11**		**MZ_{RMSSD}**		**BIS-11**		**MZ_{RMSSD}**		**BIS-11**		**MZ_{RMSSD}**	
	R	***$P_{adj.}$***	***R***	***$P_{adj.}$***	***R***	***$P_{adj.}$***	***R***	***$P_{adj.}$***	***R***	***$P_{adj.}$***	***R***	***$P_{adj.}$***
**IS**	0.155	0.547	-0.069	0.759	0.022	0.981	0.098	0.801	**-0.67**	**0.006**	**-0.755**	**0.001**
**IV**	-0.068	0.706	0.085	0.759	0.045	0.981	-0.139	0.801	0.367	0.173	**0.539**	**0.024**
**RA**	0.359	0.240	-0.14	0.759	-0.168	0.684	-0.373	0.468	**-0.606**	**0.016**	**-0.671**	**0.006**
**L5 onset**	0.237	0.547	-0.028	0.874	0.296	0.684	0.128	0.801	0.419	0.142	0.361	0.118
**M10 onset**	-0.132	0.547	0.084	0.759	-0.16	0.684	-0.044	0.890	0.372	0.173	0.416	0.080
**L5 activity**	-0.187	0.547	0.197	0.759	0.187	0.684	0.264	0.608	0.233	0.371	**0.568**	**0.021**
**M10 activity**	0.141	0.547	0.084	0.759	-0.004	0.981	-0.104	0.801	-0.288	0.273	0.177	0.442
**L5 onset (SD)**	-0.168	0.547	0.329	0.684	0.175	0.684	-0.022	0.908	0.452	0.120	**0.572**	**0.021**
**M10 onset (SD)**	-0.254	0.547	0.161	0.759	0.283	0.684	0.159	0.801	0.146	0.527	**0.492**	**0.034**
**L5 activity (SD)**	-0.152	0.547	0.086	0.759	-0.042	0.981	0.05	0.890	**0.706**	**0.0004**	**0.547**	**0.024**
**M10 activity (SD)**	-0.121	0.547	0.164	0.759	-0.12	0.791	-0.274	0.608	0.177	0.482	**0.529**	**0.024**
**RA (SD)**	-0.374	0.240	0.169	0.759	0.172	0.684	0.109	0.801	0.304	0.270	0.406	0.080

Bivariate correlations between acitgraph measure and symptoms assessed by BIS-11 and RMSSD of Mood Zoom diary entries. Significance testing performed using the False Discovery Rate to control for family-wise error (Benjamini Hochberg method) and are reported as significant a $P<0.05$ (indicated in bold).

Supplementary Table 4. NPCRA parameter partial correlations with impulsivity and mood instability

	HC				BD				BPD			
	BIS-11		MZ_{RMSSD}		BIS-11		MZ_{RMSSD}		BIS-11		MZ_{RMSSD}	
	R	$P_{adj.}$	***R***	$P_{adj.}$	***R***	$P_{adj.}$	***R***	$P_{adj.}$	***R***	$P_{adj.}$	***R***	$P_{adj.}$
**IS**	0.175	0.574	-0.108	0.679	0.013	0.948	0.113	0.839	**-0.663**	**0.012**	**-0.773**	**0.001**
**IV**	-0.096	0.607	0.156	0.677	0.031	0.948	-0.157	0.839	0.331	0.285	**0.662**	**0.008**
**RA**	0.369	0.258	-0.162	0.677	-0.192	0.710	-0.344	0.804	**-0.616**	**0.020**	**-0.694**	**0.006**
**L5 onset**	0.247	0.540	-0.04	0.905	0.313	0.710	0.084	0.839	0.403	0.174	0.357	0.160
**M10 onset**	-0.113	0.596	0.004	0.982	-0.142	0.732	-0.066	0.839	0.539	0.051	**0.553**	**0.028**
**L5 activity**	-0.204	0.547	0.256	0.677	0.187	0.710	0.222	0.839	0.269	0.318	**0.560**	**0.028**
**M10 activity**	0.139	0.547	0.105	0.679	-0.031	0.948	-0.098	0.839	-0.282	0.318	0.098	0.689
**L5 onset (SD)**	-0.144	0.547	0.249	0.677	0.234	0.710	-0.057	0.839	0.462	0.110	**0.607**	**0.018**
**M10 onset (SD)**	-0.258	0.540	0.182	0.677	0.278	0.710	0.12	0.839	0.16	0.559	0.457	0.065
**L5 activity (SD)**	-0.195	0.547	0.211	0.677	-0.032	0.948	0.022	0.839	**0.703**	**0.012**	**0.525**	**0.036**
**M10 activity (SD)**	-0.139	0.547	0.21	0.667	-0.137	0.732	-0.269	0.839	0.143	0.559	0.458	0.065
**RA (SD)**	-0.365	0.258	0.136	0.679	0.181	0.710	0.062	0.839	0.315	0.285	0.290	0.250

Partial correlations with gender and employment status inserted as covariates. Significance testing performed using the False Discovery Rate to control for family-wise error (Benjamini Hochberg method) and are reported as significant a P<0.05 (indicated in bold).

Supplementary Table 5. Group-wise comparison of partial correlation coefficients

	HC vs BD				HC vs BPD				BD vs BPD			
	BIS-11		MZ_{RMSSD}		BIS-11		MZ_{RMSSD}		BIS-11		MZ_{RMSSD}	
	**Z**	$P_{adj.}$	**Z**	$P_{adj.}$	**Z**	$P_{adj.}$	**Z**	$P_{adj.}$	**Z**	$P_{adj.}$	**Z**	$P_{adj.}$
**IS**	0.63	0.7489	-0.85	0.7353	**3.29**	**0.004**	**3.1**	**0.0096**	**2.68**	**0.0444**	**3.78**	**0.0024**
**IV**	-0.49	0.7489	1.21	0.7353	-1.49	0.1634	-2.16	0.0739	-1.04	0.4475	**-3.16**	**0.0064**
**RA**	2.36	0.2196	0.29	0.8181	**4.1**	**0.0005**	**2.7**	**0.0207**	1.98	0.1431	**2.4**	**0.0322**
**L5 onset**	-0.27	0.7872	-0.48	0.8181	-0.59	0.6057	-1.4	0.2423	-0.34	0.7339	-0.96	0.3677
**M10 onset**	0.31	0.7872	0.69	0.7353	**-2.15**	**0.0474**	-1.96	0.0856	-2.37	0.0712	**-2.52**	**0.0281**
**L5 activity**	-1.22	0.5340	-0.71	0.7353	**-2.32**	**0.0348**	**-3.03**	**0.0096**	-1.22	0.4450	**-2.35**	**0.0322**
**M10 activity**	1.25	0.5340	0.27	0.8181	**2.93**	**0.0102**	0.97	0.3472	1.8	0.1726	0.72	0.4752
**L5 onset (SD)**	-1.7	0.5340	0.23	0.8181	**-2.6**	**0.0223**	**-3.08**	**0.0096**	-1.08	0.4475	**-3.22**	**0.0064**
**M10 onset (SD)**	-0.63	0.7489	0.74	0.7353	**-3.61**	**0.0018**	-1.25	0.2817	**-3.0**	**0.0324**	-1.86	0.0944
**L5 activity (SD)**	-1.27	0.5340	-0.85	0.7353	-1.97	0.0651	-2.03	0.0848	-0.83	0.4878	-1.25	0.2536
**M10 activity (SD)**	0.49	0.7489	1.94	0.6288	-0.41	0.6818	-0.94	0.3472	-0.83	0.4878	**-2.6**	**0.0279**
**RA (SD)**	-0.7	0.7489	0.84	0.7353	**-2.5**	**0.0248**	-0.96	0.3472	-0.66	0.5556	-1.67	0.1265

Table shows Fisher Z-transformation analysis comparing the coefficients of partial correlations between rest-activity pattern parameters and symptoms between groups. P-values are adjusted using the False Discovery Rate (Benjamini Hochberg method) and are reported as significant a $P<0.05$ (indicated in bold).

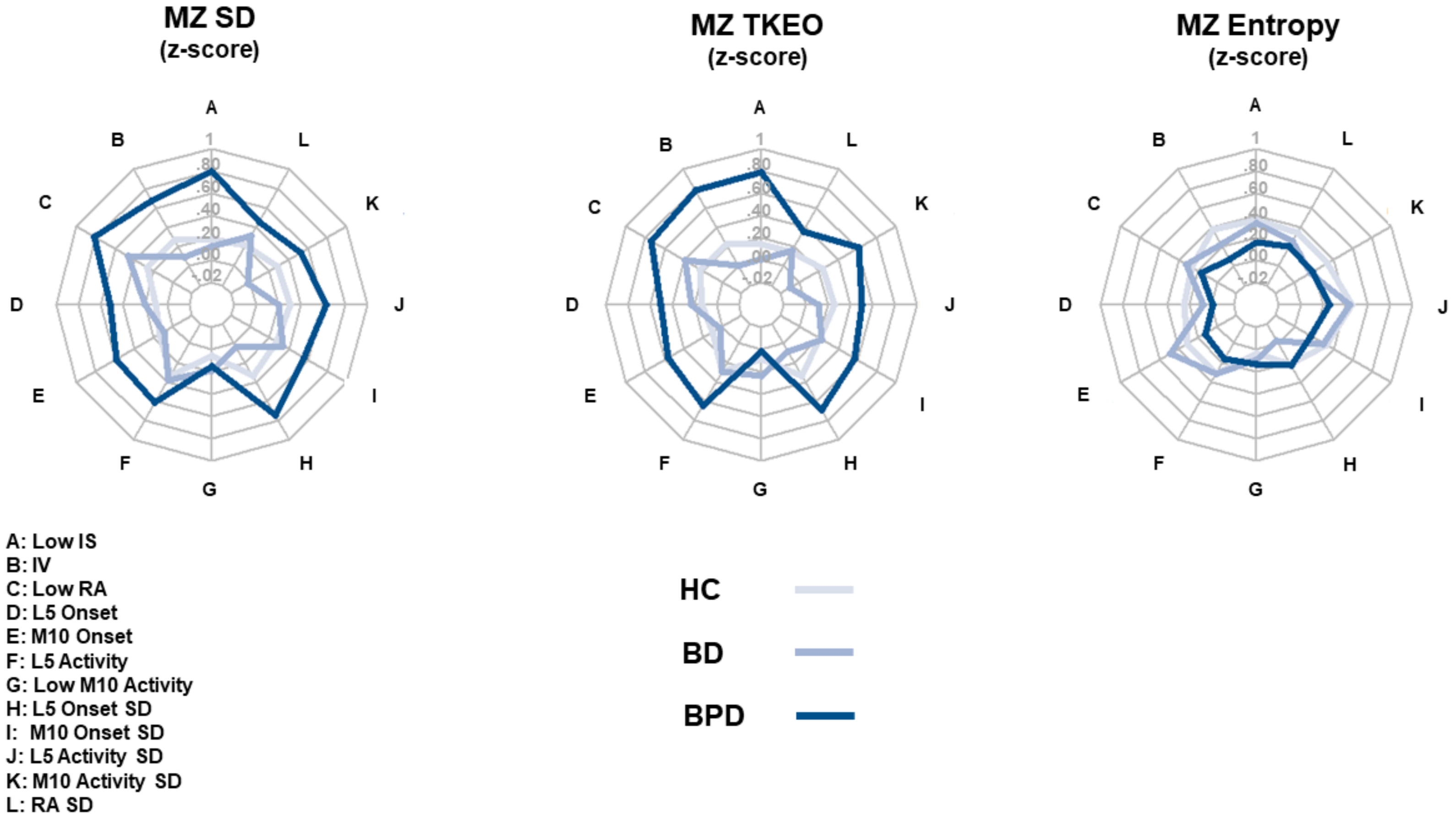

Supplementary Figure 1: Correlation between sleep/circadian rhythm parameters and MZ variability using SD, TKEO and Shannon entropy

Radar plots indicate partial correlation coefficient strength between rest-activity pattern variables and mood instability quantified using standard deviation (SD), Teager-Kaiser energy operator (TKEO) and Shannon entropy. Negative correlations (e.g. for Interdaily stability, relative amplitude, and M10 activity) were reversed and renamed for visual consistency such that higher values uniformly indicate stronger association.

Supplementary Table 6: MZ variability (SD) and NPCRA variables

	HC		BD		BPD	
	R	***P*** ***adj.***	***R***	***P*** ***adj.***	***R***	***P*** ***adj.***
IS	-0.051	0.881	0.024	0.900	**-0.769**	**0.00072**
IV	0.148	0.721	-0.059	0.832	**0.628**	**0.0119**
RA	-0.147	0.721	-0.378	0.519	**-0.781**	**0.00072**
L5 onset	-0.045	0.881	0.065	0.832	0.424	0.0842
M10 onset	-0.005	0.975	-0.059	0.832	**0.516**	**0.0408**
L5 activity	0.231	0.721	0.273	0.774	**0.548**	**0.0304**
M10 activity	0.097	0.881	-0.059	0.832	-0.016	0.947
L5 onset (SD)	0.230	0.721	-0.116	0.832	**0.700**	**0.0034**
M10 onset (SD)	0.154	0.721	0.230	0.774	0.486	0.0521
L5 activity (SD)	0.191	0.721	0.072	0.832	**0.564**	**0.0285**
M10 activity (SD)	0.169	0.721	-0.190	0.774	0.442	0.0771
RA (SD)	0.079	0.881	0.195	0.774	0.370	0.1298

Legend as in Supplementary Table 4

Supplementary Table 7: MZ variability (TKEO) and NPCRA variables

	HC		BD		BPD	
	R	***P*** ***adj.***	***R***	***P*** ***adj.***	***R***	***P*** ***adj.***
IS	-0.001	0.998	0.158	0.849	**-0.757**	**0.0016**
IV	0.095	0.998	-0.163	0.849	**0.744**	**0.0016**
RA	-0.101	0.998	-0.287	0.828	**-0.702**	**0.0032**
L5 onset	-0.013	0.998	0.092	0.849	0.418	0.0899
M10 onset	-0.052	0.998	-0.141	0.849	0.487	0.0515
L5 activity	0.180	0.998	0.177	0.849	**0.587**	**0.0199**
M10 activity	0.062	0.998	-0.113	0.849	0.143	0.5603
L5 onset (SD)	0.224	0.998	-0.063	0.849	**0.633**	**0.0108**
M10 onset (SD)	0.081	0.998	0.096	0.849	**0.501**	**0.0498**
L5 activity (SD)	0.132	0.998	-0.034	0.916	0.424	0.0898
M10 activity (SD)	0.112	0.998	-0.282	0.828	**0.555**	**0.0273**
RA (SD)	0.006	0.998	0.021	0.916	0.255	0.3182

Legend as in Supplementary Table 4

Supplementary Table 8: MZ variability (Entropy) and NPCRA variables

	HC		BD		BPD	
	R	***P*** ***adj.***	***R***	***P*** ***adj.***	***R***	***P*** ***adj.***
IS	-0.256	0.426	-0.217	0.598	-0.024	0.955
IV	0.278	0.426	0.091	0.801	-0.083	0.955
RA	-0.146	0.568	-0.212	0.598	-0.025	0.955
L5 onset	0.116	0.575	-0.083	0.801	-0.183	0.955
M10 onset	0.195	0.426	0.398	0.377	-0.02	0.955
L5 activity	0.201	0.426	0.207	0.598	0.028	0.955
M10 activity	0.117	0.575	0.058	0.833	0.014	0.955
L5 onset (SD)	0.082	0.657	-0.196	0.598	0.099	0.955
M10 onset (SD)	0.217	0.426	0.180	0.598	0.026	0.955
L5 activity (SD)	0.37	0.426	0.350	0.377	0.14	0.955
M10 activity (SD)	0.231	0.426	0.025	0.898	0.056	0.955
RA (SD)	0.24	0.426	0.139	0.707	0.068	0.955

Legend as in Supplementary Table 4

Supplementary Table 9: Discriminant properties of actigraph measure for diagnosis using ROC curve classification analysis

	HC vs BD						HC vs BPD						BD vs BPD					
	**AUC**	***Sens.***	***Spec.***	***Cut off***	***PPV***	**NPV**	**AUC**	***Sens.***	***Spec.***	***Cut off***	***PPV***	**NPV**	**AUC**	***Sens.***	***Spec.***	***Cut off***	***PPV***	**NPV**
**Low IS**	0.343	0.97	0.029	0.59	0.48	0.50	0.452	0.06	0.94	0.15	0.36	0.54	0.624	0.52	0.77	0.3595	0.61	0.71
**IV**	0.265	0.09	0.853	1.37	0.38	0.51	0.317	0.24	0.79	1.23	0.42	0.63	0.545	0.86	0.29	0.716	0.46	0.77
**Low RA**	0.488	0.97	0.147	0.90	0.51	0.83	0.655	0.86	0.5	0.83	0.47	0.82	0.679	0.76	0.58	0.809	0.55	0.78
**L5 onset**	0.524	0.97	0.029	20:12	0.48	-	0.833	0.71	0.85	01:35	0.75	0.83	0.789	0.71	0.81	01:37	0.71	0.81
**M10 onset**	0.655	0.77	0.59	09:35	0.61	0.69	0.693	0.86	0.62	10:00	0.58	0.88	0.584	0.67	0.58	11:37	0.28	0.48
**L5 activity**	0.483	0.74	0.353	4.54	0.5	0.58	0.574	0.33	0.88	8.97	0.64	0.68	0.598	0.48	0.74	6.05	0.56	0.68
**Low M10 activity**	0.533	0.94	0.206	89.82	-	0.52	0.618	0.95	0.35	68.42	0.48	0.92	0.625	0.91	0.39	59.92	0.5	0.86

AUC determined by inspecting ROC curve. Optimal sensitivity and specificity and resultant cut-off scores determined by calculating the Youden Index which designates the maximum value of sensitivity + specificity - 1 along points of the ROC curve. AUC; area under the curve, Sens.; sensitivity, Spec.; specificity, PPV; positive predictive value, NPV; negative predictive value. Dashed line for PPV or NPV indicates no cases were identified using that cut-off.
